# Internal Hernia as a Rare Complication of Acute Appendicitis

**DOI:** 10.7759/cureus.56613

**Published:** 2024-03-21

**Authors:** Omar M AlSarraj, Awadh Alqahtani, Faisal S Alqahtani, Srikar Billa, Mohammed AlMayouf

**Affiliations:** 1 Department of Surgery, Sulaiman Al-Habib Hospitals, Riyadh, SAU; 2 Department of Surgery, King Saud University, Riyadh, SAU; 3 College of Medicine, Imam Abdulrahman Bin Faisal University, Dammam, SAU; 4 College of Medicine, Prince Sattam Bin Abdulaziz University, Alkharj, SAU

**Keywords:** clinical appendicitis, diagnosis of appendicitis, imaging in appendicitis, appendictis, atypical appendicitis

## Abstract

Appendicitis is a well-known and highly common surgical emergency disease, yet it presents with a wide variety of manifestations. This is a case report of a 47-year-old female who presented with a complaint of having constant crampy right lower abdominal pain for two weeks. The patient reported having a sudden onset of symptoms that went with the typical picture of acute appendicitis that occurred two weeks ago. Our pre-op workup was inconclusive; therefore, we planned to go for a diagnostic laparoscopy, where surprisingly, the appendix was long, inflamed, and attached to the posterior wall of the cecum. Thus, a ring-like structure was developed, in which 8 to 10 cm of the terminal ileum (the last part of the small bowel) was going through and causing an internal hernia. Although blood and radiology workups provide valuable assistance in diagnosing common cases, a highly suspicious sense and skillful surgeons with good clinical experience play a major role in managing such rare presentations.

## Introduction

Appendicitis is the most common surgical emergency in the world [1]. About 7%-12% of the general population is affected [1,2], whereas approximately 16% of people in the Western world experience an appendicectomy at some stage during their life [3]. Added to that, an average of 250,000-300,000 cases of acute appendicitis are diagnosed annually in the United States. Appendectomy surgery is considered a straightforward procedure for new surgical residents to learn proficiently during training [4,5]. However, appendicitis is an easy diagnosis to miss, and only half of the appendicitis patients present typically, with periumbilical pain followed by nausea, vomiting, and migration of the pain to the right lower quadrant (which we call the classic picture of acute appendicitis) [6]. Moreover, especially in premenopausal women, making the diagnosis is particularly challenging because many diseases can mimic appendicitis, such as torsion of the ovary, pelvic inflammatory disease, and ectopic pregnancy [6]. Lastly, sometimes appendicitis patients will present with complications or consequences of the inflamed appendix, not the direct presentations that we usually see in emergencies. This study aims to report the presentation and management of a middle-aged female patient coming with the presentation of an atypical complication of appendicitis, that is, small bowel obstruction due to an internal hernia.

## Case presentation

The patient is a 47-year-old female, known to previously have adenomyosis, which resulted in a total hysterectomy with bilateral salpingectomy two years ago. Added to that she underwent a laparoscopic sleeve gastrectomy six years ago, and she has maintained her body mass index (BMI) within the normal range (BMI 23). She presented to our general surgery clinic with a complaint of constant cramping and right lower abdominal pain that started two weeks ago. The patient provided a history of having acute generalized abdominal pain, sudden onset two weeks ago. It was all over the abdomen then shifted to her right lower side, with a positive history of nausea, vomiting, and subjective fever. She sought medical care in a dispensary center near her house, where they did an abdominal ultrasound and found a borderline appendix diameter (0.8 cm) with free fluid in the pelvis. They planned to refer her to an emergency department because she needed a computed tomography (CT) scan of her abdomen and pelvis to rule out appendicitis. She said she felt fine and didn’t need to go.

**Figure 1:**
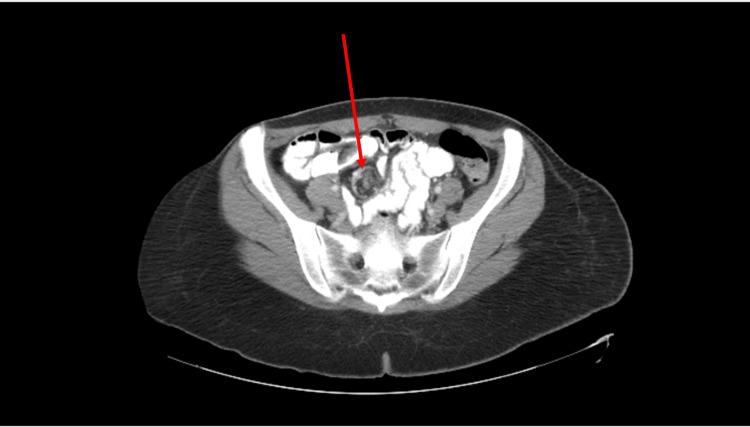
CT scan of the abdomen and pelvis with intravenous contrast showing an appendicular mass with a whirl sign. CT, computed tomography

**Figure 2:**
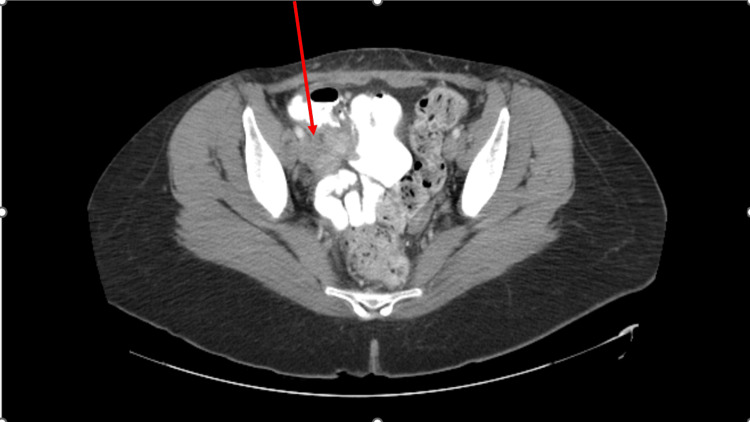
CT scan of the abdomen and pelvis with intravenous contrast showing an appendicular mass. CT, computed tomography

She did not seek any additional medical care until she came to our clinic two weeks after that event. Reviewing her complaint, we found that she was feeling nauseated, with a few episodes of post-prandial vomiting, especially after heavy meals. In addition to mild abdominal distension, there was a loss of appetite and a decrease in the frequency of her bowel movements; however, she was not constipated. Upon examination, we found the abdomen was soft and lax with mild tenderness upon palpation of McBurney’s point. The rest of the examination revealed no significant findings. So we decided to do a CT scan of the abdomen and pelvis with intravenous and oral contrast to rule out appendicular mass or other pathology. The report came back with a right adnexal enhancing mass with no conclusive features and a query whirl sign over the same area (Figures 1-2). Blood work came back inconclusive too; the patient’s inflammatory markers (C-reactive protein [CRP] and erythrocyte sedimentation rate [ESR]) were normal. Also, her complete blood count results ruled out leukocytosis. Therefore, we planned to perform diagnostic laparoscopic surgery due to the ongoing pain. 

**Figure 3:**
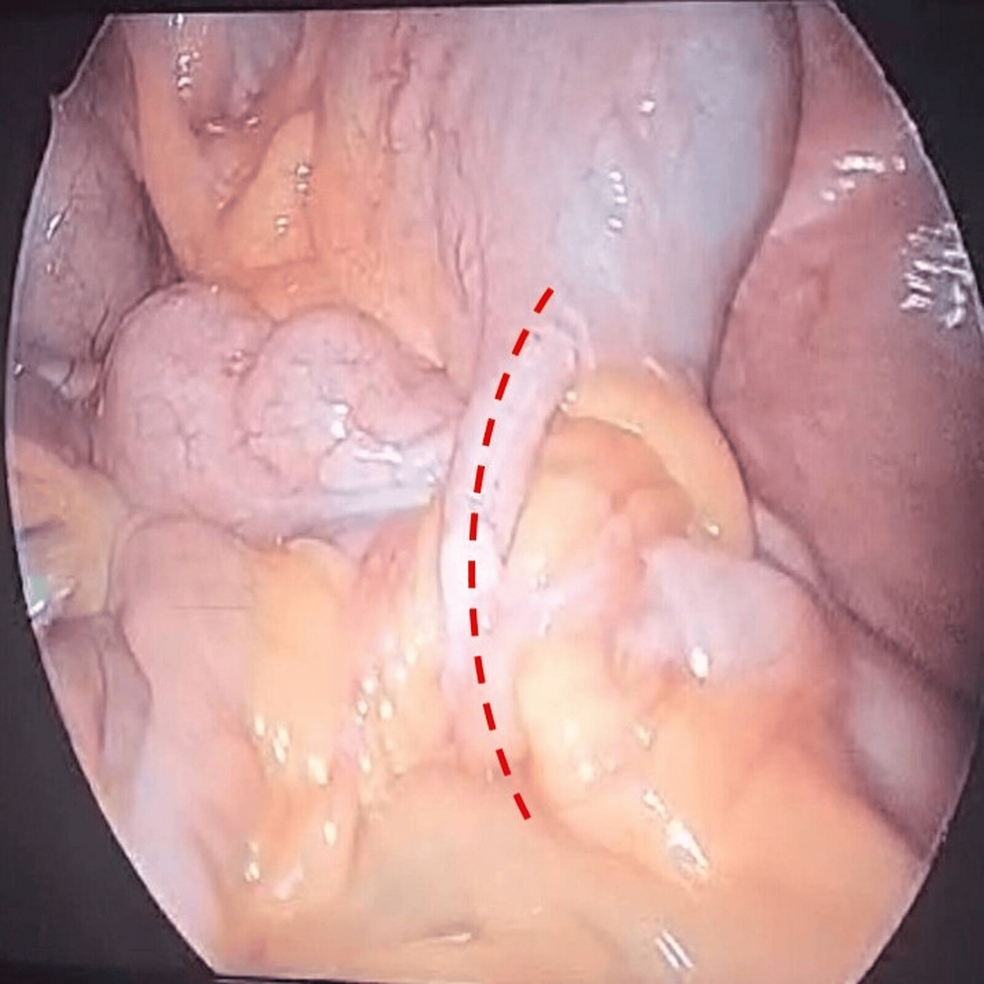
Long appendix (dashed red line) forming a ring-like structure with the small bowel passing through, causing the internal hernia.

**Figure 4:**
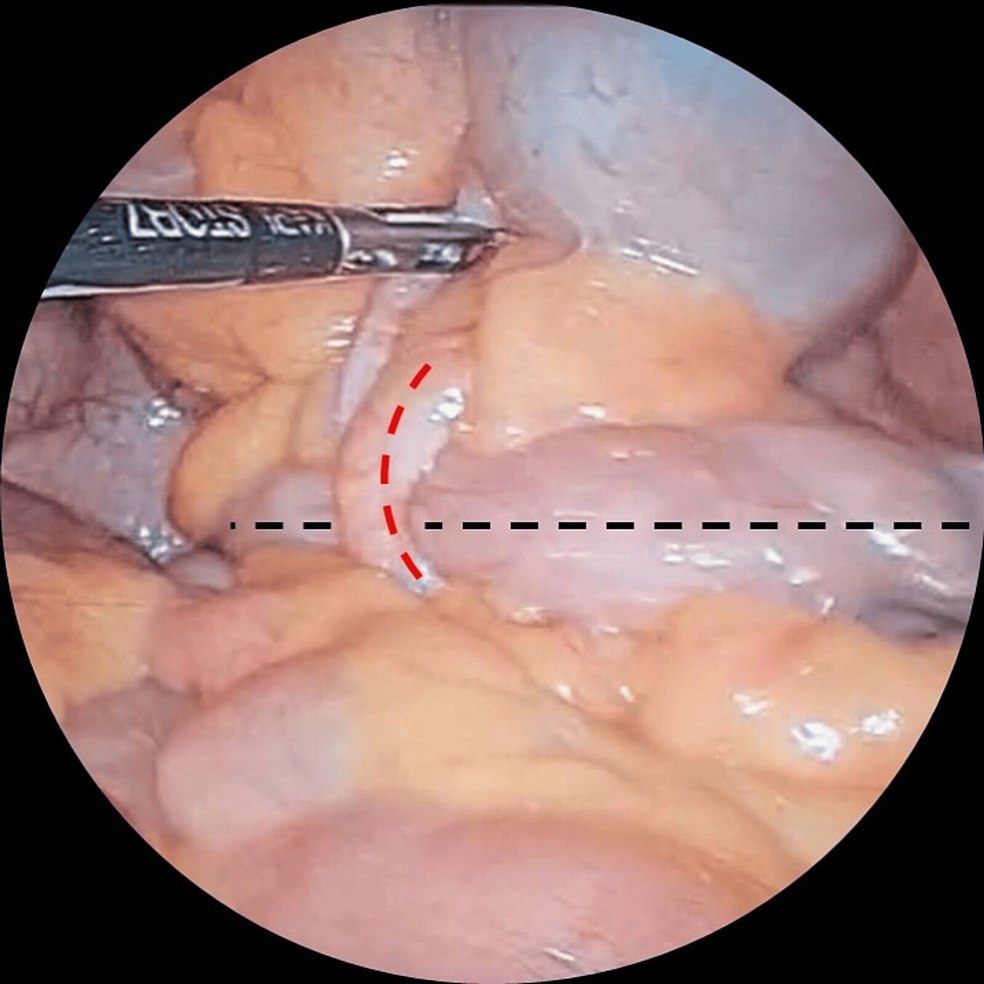
Long appendix forming a ring-like structure (dashed red line) with the small bowel passing through (dashed black line), causing the internal hernia.

**Figure 5:**
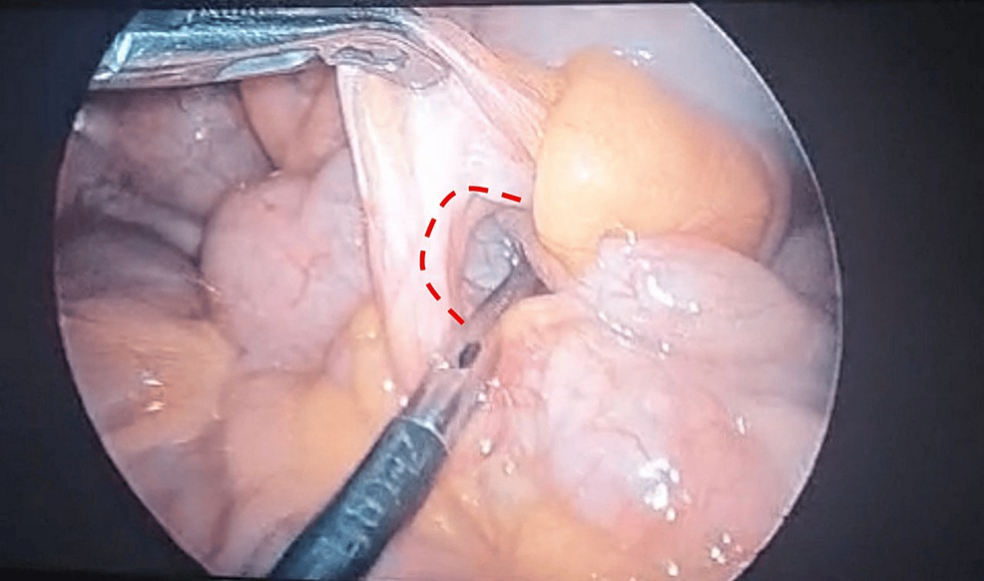
Half of the ring-like structure is formed by the long appendix (dashed red line), and the other half is retrocecal and cannot be visualized.

The patient was cleared by anesthesia as per routine. A prophylactic antibiotic (2 g of cefazolin, which is second-generation cephalosporin) was given upon induction of general anesthesia. The patient lay on the table in the supine position and was prepped and draped under usual sterile conditions. Her abdomen was insufflated using a 5 mm vesiport through an optical maneuver. Then other ports were inserted under direct vision. After adhesions were released, the cecum was identified and tracked until its base and the appendix were visible. Surprisingly, the appendix was long and inflamed and attached to the posterior wall of the cecum. It made a ring-like structure where about 8 to 10 cm of the terminal ileum (the last part of the small bowel) went through, causing an internal hernia (Figures 3-5). Hernia reduction was performed, and then the appendix was freed, with its mesentery controlled using a LigaSure vessel sealing device, then divided with a stapler, and finally removed with an endobag. The patient was extubated, shifted to recovery, and then moved to her room, as per routine. The postoperative period was uneventful, and the patient was discharged on postoperative day 1. She was free from complaints at the outpatient follow-ups. The histopathology report revealed acute and subacute appendicitis with periappendicitis, with no evidence of malignancy.

## Discussion

The diagnosis of a patient with acute abdominal pain, suspected of having appendicitis, remains multifactorial and still relies to a large extent on surgeon experience. In atypical presentations of appendicitis, further investigations such as ultrasound, CT, and even laparoscopy may be used [3]. The complications of appendicitis are divided into two groups. The first one includes highly common complications such as perforated appendicitis with generalized peritonitis, appendiceal mass, appendiceal abscess, and sepsis [7]. The second includes uncommon complications such as peritoneal adhesion formation and small bowel intestinal obstruction [7]. The last is mostly known to obstruct adhesion. Few cases of mechanical small bowel obstruction (secondary to internal hernia) developed as a direct result of acute appendicitis have been reported in the literature [8].

The definition of internal hernia is a protrusion of abdominal viscera, most commonly small bowel loops, through a peritoneal or mesenteric opening into a compartment in the abdominal and pelvic cavity. This can be divided into congenital and acquired subtypes [9]. The acquired one, which is our topic here, has three common reasons, which are inflammation, trauma, and previous surgery, such as bariatric surgeries [10]. The chance of having abdominal internal hernias is rare, and according to the classification of internal abdominal herniations devised by Ghahremani [11] can be separated into six main groups: paraduodenal hernias (50%-55% of internal abdominal herniations), hernias through the foramen of Winslow (6%-10%), transmesenteric hernias (8%-10%), pericecal hernias (10%-15%), intersigmoid hernias (4%-8%), and paravesical hernias (<4%) [12]. 

Regarding the clinical presentations of pericecal hernias, which is the case in our scenario, there is a wide variety. Symptoms may range from nonspecific, such as nausea, vomiting, and generalized abdominal pain, to more classic symptoms characterized by episodes of intense postprandial lower abdominal pain, predominantly over the right lower quadrant area. These symptoms can be very similar to appendiceal pain, as observed in our patient presentation [11]. As in all types of internal hernias, the risk of bowel strangulation and ischemia is always there. It is reported that patients with pericecal hernias can rapidly progress to strangulation, and the mortality rate is as high as 75% [10]. Therefore, diagnosing the cause and initiating prompt surgical intervention is obligatory to prevent any delay that may increase patient mortality.

This point is worth mentioning, even high-sensitivity radiological investigations like CT scan, which has a fundamental part in the diagnostic workup, may not be conclusive in picking up such cases. Therefore, high numbers of atypical presentations of appendicitis will be diagnosed and confirmed only by diagnostic laparoscopy, which has a beneficial role in both diagnostic and therapeutic purposes [13,14]. To sum up, picking up an atypical presentation of appendicitis and diagnosing it with the aid of a radiological workup is still not easy and straightforward. Thus, the decision to push the patient to the operating theater for diagnostic laparoscopic surgery still requires experience and wisdom from the surgeon. 

## Conclusions

Appendicitis, a widely recognized surgical emergency, exhibits considerable variability in its presentation. Uncommonly, it can manifest as an internal hernia leading to intestinal obstruction, presenting a diagnostic challenge even with high-sensitivity radiological investigations such as CT scans. Hence, a keen sense of suspicion coupled with extensive clinical experience becomes paramount in managing such cases effectively. The decision to proceed with surgical intervention must be made judiciously and promptly, as life-threatening complications such as bowel ischemia can ensue if there is any delay.
